# Supplementary figures and images for: The haemodynamics of the human placenta in utero
Source: PLoS Biol. 2020 May 28;18(5):e3000676. doi: 10.1371/journal.pbio.3000676 (PMC7255609; doi:10.1371/journal.pbio.3000676)

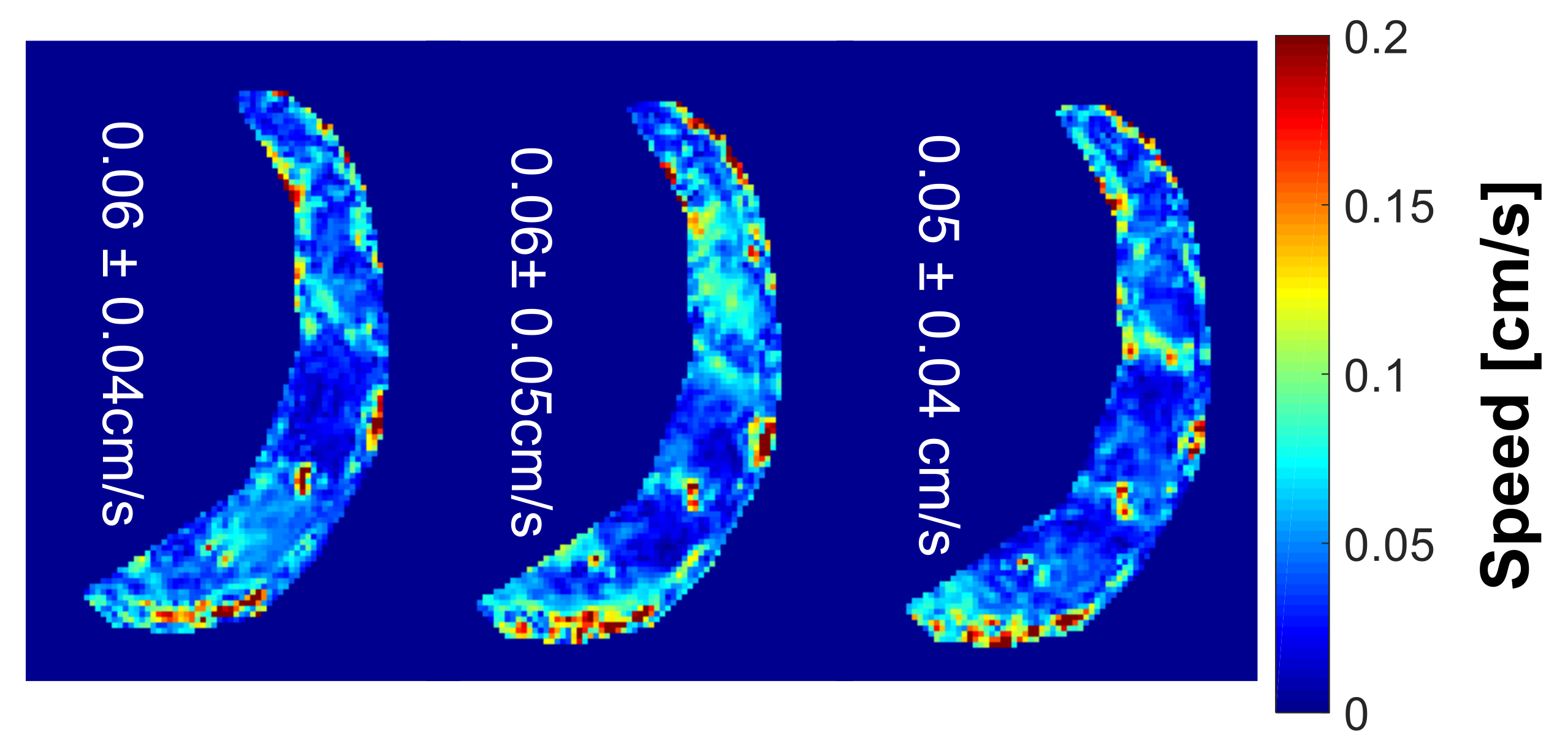

Supplement: S1 Fig — The average net speed between the scans was 0.0577 ± 0.005 cm/s. HC, healthy control pregnancy. (TIF) [file pbio.3000676.s006.tif]

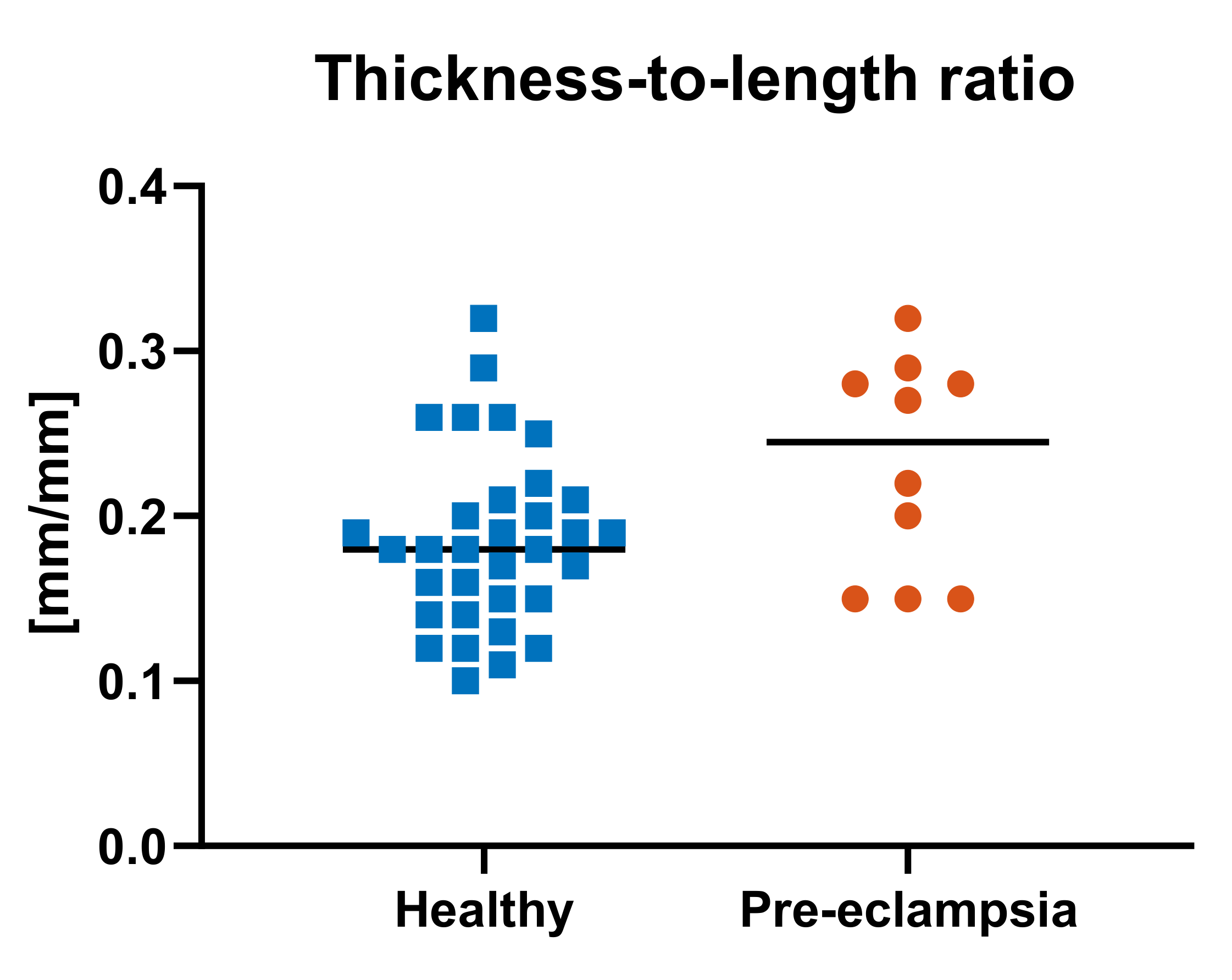

Supplement: S2 Fig — Underlying data are provided in S1 Data. (TIF) [file pbio.3000676.s007.tif]

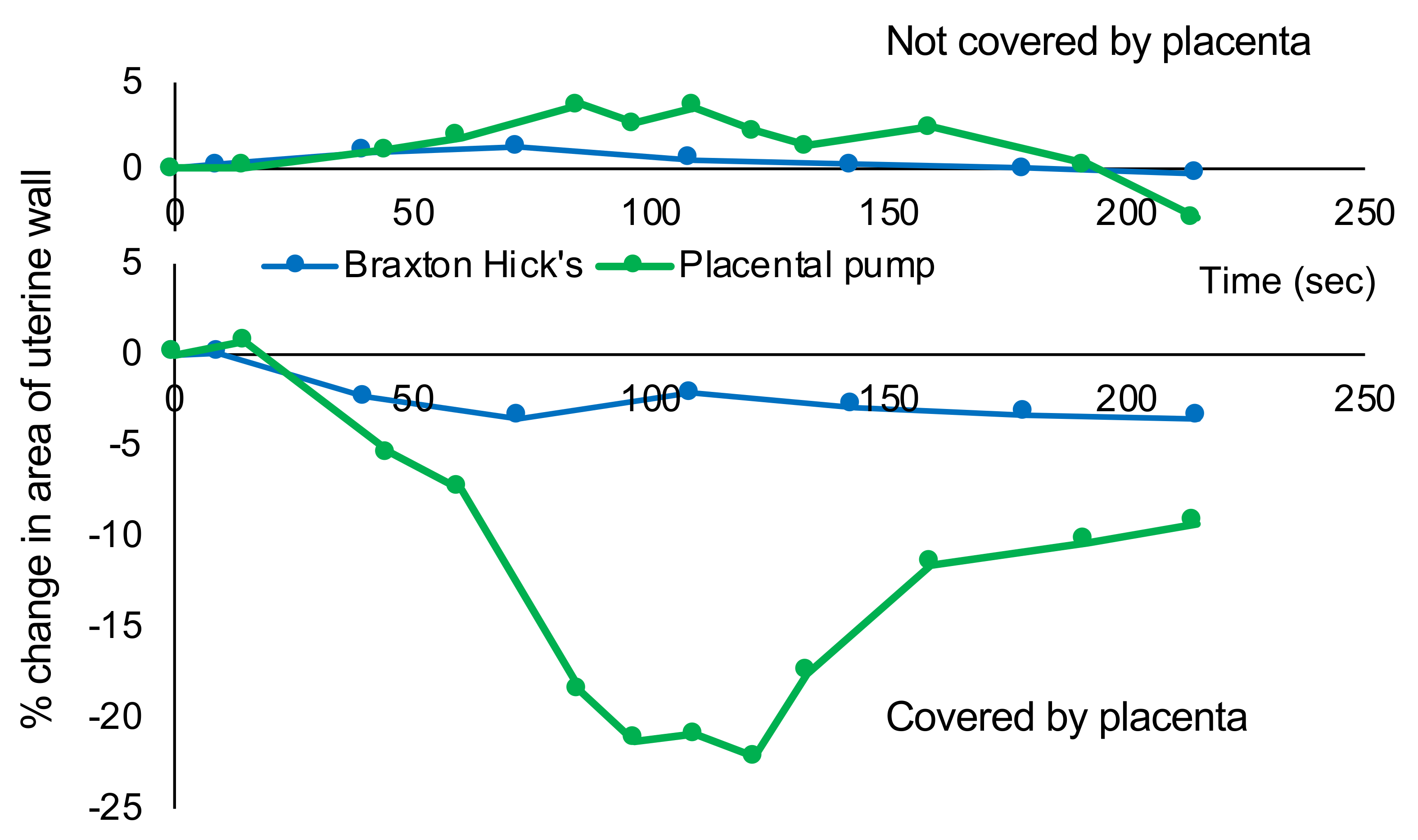

Supplement: S3 Fig — Time course of contractions in the same participants as S1–S4 Movies, with each line representing the change (%) of the wall not covered by placenta (increased during contraction) and the area of the uterine wall covered by placenta (decreased during contraction). Underlying data are provided in S1 Data. HC, healthy control pregnancy. (TIF) [file pbio.3000676.s008.tif]

Decidual  
surface

Infarct

Chorionic  
villi

5 mm

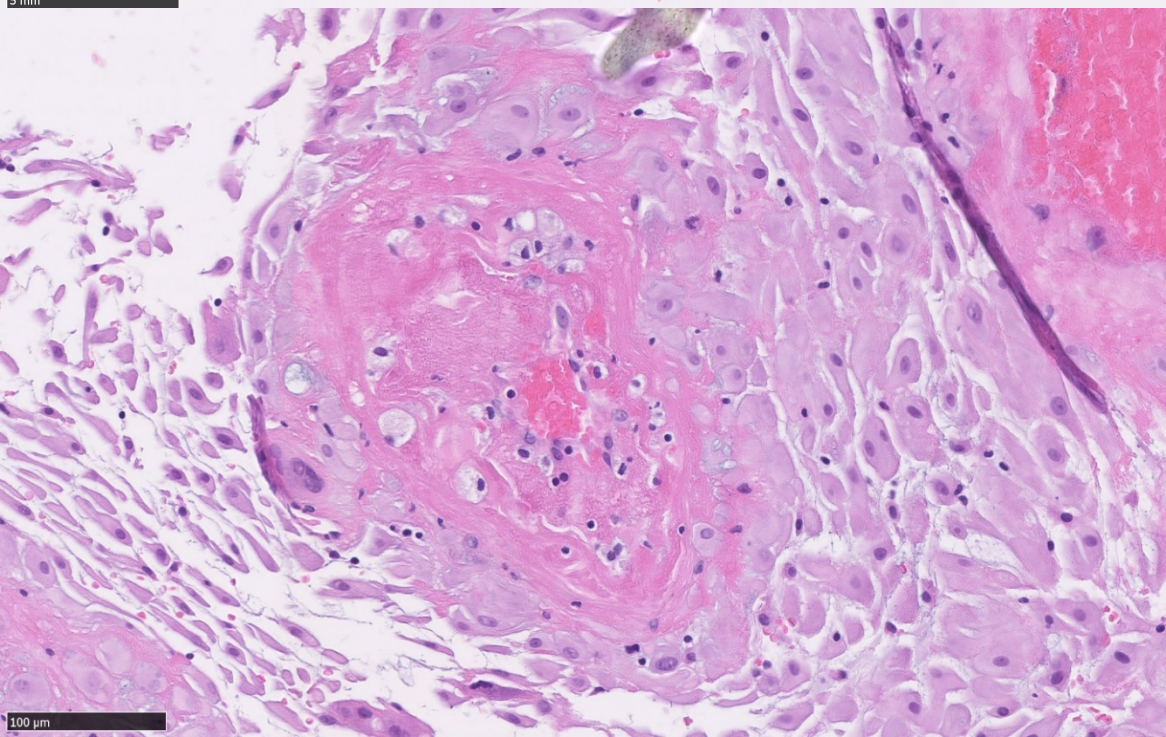

100  $\mu$ m

Supplement: S4 Fig — Haematoxylin and eosin staining from a placenta of a patient diagnosed with severe preeclampsia and HELLP syndrome and delivered by emergency cesarean section at 28 + 6 weeks’ gestational age. A low-magnification overview of the tissue (a, scale bar 5 mm) and a higher-magnification image (b, scale bar 100 μm). This image demonstrates decidual arteriopathy including focal atherosis in keeping with a histological diagnosis of maternal vascular malperfusion. Placenta examined as per ‘Tissue pathway for histopathological examination of the placenta’ Royal College of Pathologists, 2017. PE, preeclamptic pregnancy. (PDF) [file pbio.3000676.s009.pdf]

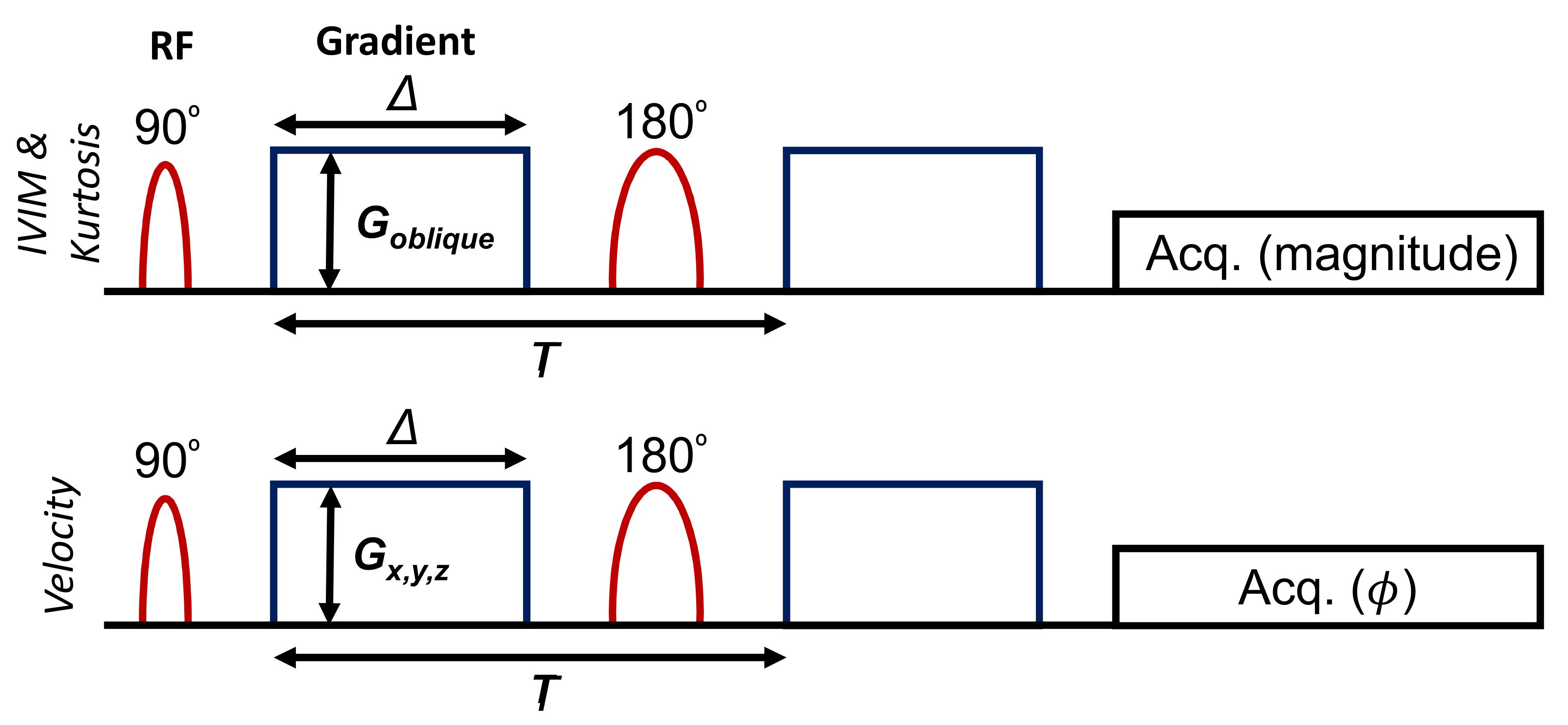

Supplement: S5 Fig — fIVIM and kurtosis: Δ, the duration of gradient lobes, and T, the time between the start of each gradient lobe, are fixed (see S3 Table). The height of Goblique is increased to increase the b-value. After the second gradient, the magnitude of the MR signal is acquired. Velocity: Δ and T have different lengths compared with the fIVIM and kurtosis acquisition (see S3 Table). The gradient height is set to 21 mT/m for maximum velocity encoding of ±0.5 cm/s. After the second gradient, the MR phase signal (Φ) is reconstructed for velocity measurement. fIVIM, intravoxel incoherent motion fraction; MR, magnetic resonance; PGSE, pulsed gradient spin echo. (TIF) [file pbio.3000676.s010.tif]

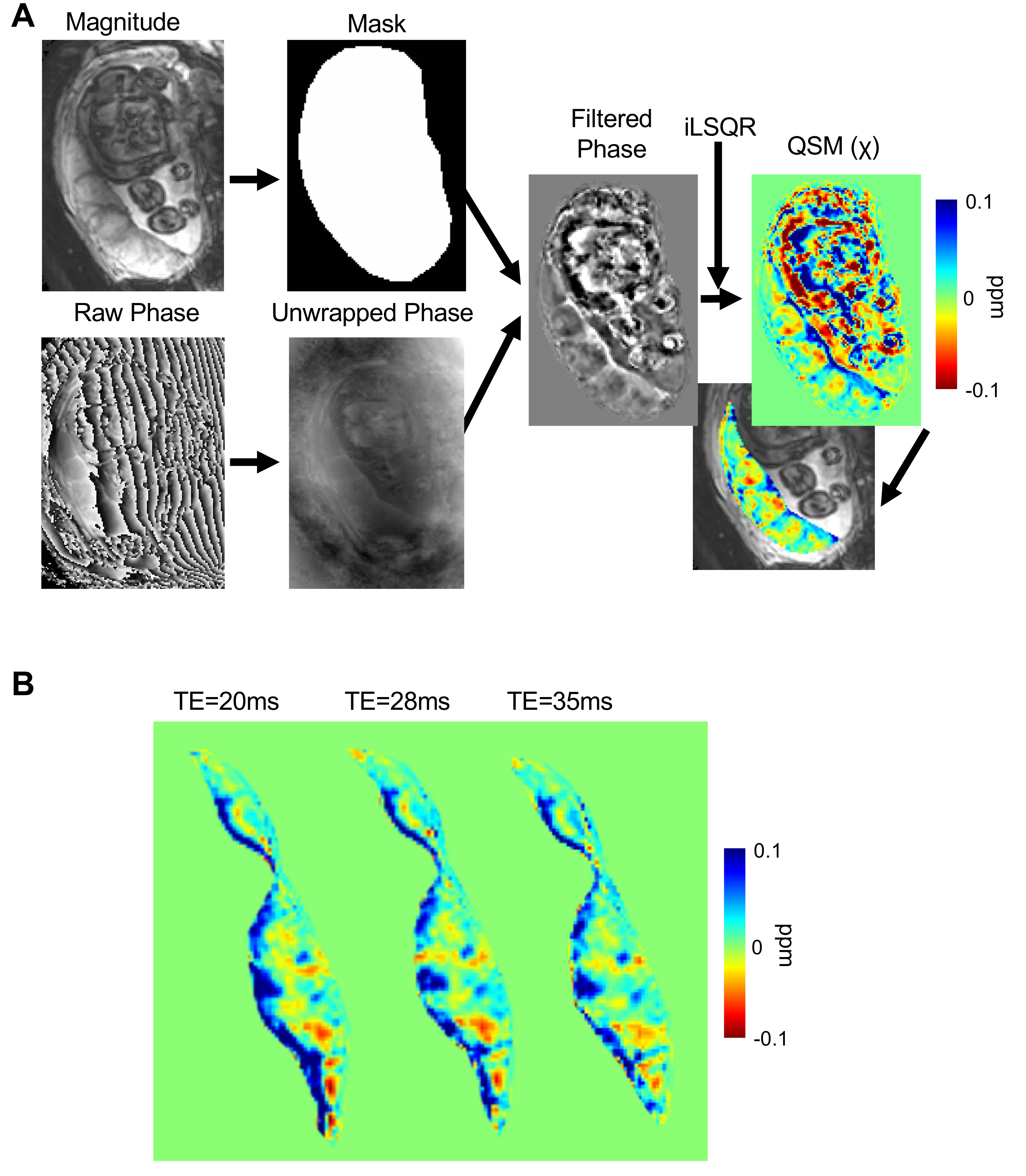

Supplement: S6 Fig — (A) First column: The raw magnitude and phase data acquired from the gradient echo acquisition (S3 Table). Second column: The uterus is masked in the magnitude data, and phase images are unwrapped within that mask using a Laplacian-based method. Third column: A 2D V-SHARP filter slice by slice to remove the background phase is applied to the unwrapped phase, and subsequently the uterus mask is applied to make the filtered phase. Fourth column: Susceptibility maps are calculated using LSQR [70]. (B) The χ calculated using the QSM pipeline for three different TE values shows its general insensitivity to the TE used in the gradient echo EPI acquisition. EPI, echo-planar imaging; QSM, quantitative susceptibility mapping; TE, echo time. (TIF) [file pbio.3000676.s011.tif]
