# Supplementary material for: The haemodynamics of the human placenta in utero
Source: PLoS Biol. 2020 May 28;18(5):e3000676. doi: 10.1371/journal.pbio.3000676 (PMC7255609; doi:10.1371/journal.pbio.3000676)
Supplement: Attachment — Submitted filename: Response to reviewers Gowland.docx [file pbio.3000676.s017.docx]

**Response to the Editor**

We would like to thank you for your own very thoughtful response, and for giving us the opportunity to respond to the reviewers’ comments.

As we have explained in the paper, the function of the human placenta can only properly be studied in utero in pregnant women. This puts practical limits on experimental design but we hope that the reviewers will agree that our data provides new insights into, and substantiates previous hypotheses about, the function of the human placenta.

Of particular note, two of the reviewers note that the comparison between normal and abnormal pregnancies is difficult to parse given the range and lack of characterization of the abnormal pregnancies.

We agree that the patient group was too heterogeneous and so now we have restricted our compromised group to women with pre-eclampsia. We did consider removing the compromised pregnancies and would still be happy to do so but we think that the compromised pregnancy data included in a revised format does add to the work.

**Reviewer #1:**

We agree with the reviewer that the area of research is of great importance to human pregnancy, and thank them for their comments that the results are interesting. We hope that they agree with us that this paper has developed as a result of the extra data that we provide and new, more rigorous analyses we have performed on venous outflow. We have attempted to increase the level of detail in the manuscript in line with all the reviewers’ comments and justify any correlations we have made.

R1.1 The study is based on pathologies (preeclampsia and fetal growth restriction), which in many cases could be markedly different. This may lead to data misinterpretation and to increased variance (e.g., Fig. 4B), and weakly supported conclusions.

We thank the reviewer for this comment, and have amended the manuscript to include healthy controls (HC) and women with preeclampsia (PE) only. The preeclampsia group includes women with babies that are normally grown or growth restricted. As the reviewer predicted, despite smaller numbers this has generally increased separation between groups (e.g. flow in the placenta and chorionic plate Figure 2b).

R1.2 The author should have supported their data by histopathological correlations.

Standard clinical care locally requires that all placentas from compromised pregnancies are sent for histology, and yet in the heat of an emergency delivery this does not always happen. Histological diagnosis, where available has been included (Table S2a) and the placental pathology has been confirmed by an independent pathologist (Dr Craig Platt) who reviewed the slides and is included as a co-author. We have not performed histological correlations since we do not have quantitative histological data but this would be important future work, particularly in the analysis of the kurtosis data. We have added a comment to this effect on pages 6 and 12.

R1.3 While the authors make a strong point about venous return flow, the supporting data are scarce. Venous outflow speeds are conjectured, but not tested.

We thank the reviewer for this comment. We have now undertaken an additional analysis and added a new figure (Fig. 2e&f and text on page 4), which demonstrates that the movement at the basal plate interface of the placenta is faster perpendicular to the basal plate – in both the inward and outward directions – than along it, and that there was a trend for this difference to be even faster in preeclampsia. This indicates flow into and out of the placenta.

R1.4 The concept of placental contractions is very interesting, but not well proven. In Fig. 5, the authors note areas of myometrial thickening under the placenta, suggestive of regional uterine, not placental contraction.

We probably confused matters by naming this phenomenon the ‘placental pump’. What we have observed is a thickening and shortening of the uterine wall under the placenta (whilst the rest of the uterus actually expands) and a simultaneous reduction in the volume of the placenta. We have presented the data for this in Figure 5 which shows the wall contracting under the placenta but not across the whole uterus. In order to illustrate the phenomenon more clearly we have added movies showing the contraction (Figure S3a-d). We hope that we have more clearly described this phenomenon in the revised manuscript on page 9 and 11, and renamed it the ‘uteroplacental pump’. We have also identified a Braxton Hicks contraction in the dataset and found that that has no significant effect on the placental volume (Figure S3e).

R1.5 The interpretation of Fig. 2C in the text (page 4, lines 15-18) is entirely unclear, and does not conform the hypothesis stated within.

We think that the text was confusing because we had unfortunately completely failed to discuss the chorionic plate. We have now added an additional reference and a further sentence about the chorionic plate on page 4 and hope the text is now clear.

R1.6 The presentation of the figures is commonly not sufficiently informative. Fig. 1 does not enhance the text. In Fig. 2B the red and blue arrows may be missing, and there are no text results associated with Fig 2b.

We feel Figure 1a is necessary since in our experience many biologists, clinicians and imaging scientists are unaware of the basic structure of the placenta. We could move it to supplementary materials if the reviewer and editor prefer. However to further enhance interpretation we have now combined it with the previous Fig 2b, which indicates the regions of interest used in this study. We have now referred to Figure 1b (old Figure 2b) in the text on page 4.

**Reviewer #2: Ananth Karumanchi, signed review**

These are very difficult studies and I commend the authors for this work.

We thank the reviewer for understanding the complexity of these in utero human studies.

R2.1 Given PLOS biology is a general biological journal, many people may not be familiar with the MRI language. Perhaps a glossary of the some of terms used would be very helpful

Thank you for this sensible idea. We have now included this in Table S1 which is referred to on page 3.

R2.2 Maternal oxygenation: I would like to see more data/figure on oxygenation studies in HP and CP. I could tell from figure 4B what is the interpretation. Are the placentae of compromised pregnancy equally oxygenated as healthy pregnancies? Authors state that compromised pregnancies average placental susceptibility was higher and more variable? This seemed confusing to me - susceptibility to what?? I would like to see oxygenation data for healthy and compromised pregnancies that is easy for readers to follow.

The term magnetic susceptibility is the terminology used in physics to quantify how much a material will become magnetized in an applied magnetic field. This most obviously depends on iron content but it also depends on the oxidation state of the iron, so in this context it depends on the haemoglobin oxygen saturation. What figure 4a illustrates and 4b shows is that in the healthy placenta the whole placenta is well oxygenated, whereas in the compromised pregnancy the oxygenation level is lower and varies considerably. If the placenta consisted only of adult blood a susceptibility of -0.1 would correspond to fully oxygenated blood and a susceptibility of +0.3 would correspond to a saturation of about 88% [1]. However these values will overestimate the oxygenation since the placenta also contains villous tissue with a susceptibility of approximately zero, and the sensitivity of fetal blood susceptibility to deoxygenation is 75% that of adult blood [2,3]. We have now added more information into the text on page 12.

R2.3 Placental pump - what is the significance of these in complicated pregnancies.

At this stage we cannot answer this question and are loathed to speculate too far. From the raw data it seems that the contractions are more common in compromised pregnancies but at this stage we cannot say if this is a significant difference, in part due to the limited time over which we are able to scan pregnant women. We have added text to make this clear on page 9 and 11.

**Reviewer #3: Dr. John Kingdom, Mount Sinai Hospital, University of Toronto, Canada. Signed review.**

R3.1 The investigators report T2* MRI findings in a cohort of normal pregnancies (strong) and a group of rather ill-defined abnormal pregnancies (weak).

We hadn’t previously reported T2* but we have now included this as it is becoming a standard assessment of fetal compromise [4]. There is a striking separation of the PE and HC pregnancies and interestingly a variation in T2* with birthweight even for the HCs (Fig 4d) (text added on page 8). T2* is affected by tissue oxygenation but the relationship is complex (see page 8 also).

We have characterised the compromised pregnancies more fully and have only included women with a diagnosis of pre-eclampsia in the compromised group. Further characterisation (see Table S2a) includes available first and second trimester placental markers (PAPP-A, HCG, AFP), blood pressure, proteinuria levels, medication, placental thickness, and volume at the time of MRI and delivery, birth weight and centile, and histological diagnosis where available. For all subjects placental volume to thickness ratio (at MRI) is plotted in Fig S2 (page 10; raw data in Table S2a, demographics and clinical worksheets). We could add the placental volume/estimated weight at time of MRI to table S2a if the reviewer thinks that would be helpful. Furthermore as mentioned above, placental T2* is plotted in Fig 4d, discussed on page 9.

R3.2 Surprisingly, there is no maternal-fetal medicine (high-risk Obstetrics) involvement.

Dr Nia Jones in a Clinical Academic at the University of Nottingham and a Consultant Obstetrician and a sub-specialist in feto-maternal medicine.

R3.3 There is also no reported placental pathology.

We have now added the data to the cases of pre-eclampsia where available (see Table S2a and Figure S4). We have also discussed this on page 12.

R3.4 Therefore the "compromised" pregnancies are poorly phenotyped. The authors focus on utero-placental perfusion and oxygen delivery, but do not show any uterine artery Doppler; nor do they verify (by pathology) that the pregnancies had impaired utero-placental perfusion (expressed as maternal vascular malperfusion [MVM] pathology) - as opposed to either no specific disease (constitutionally-small) or rare perfusion-independent diseases that are expressed as preeclampsia and/or fetal growth restriction. There are no data on circulating angiogenic growth factors (for example placenta growth factor [PlGF]) which is established (Benton S et al., publications) to provide independent verification of placental pathologic diseases. Therefore, the reader is unaware what if any underlying placental disease is being studied in this small heterogeneous group of abnormal pregnancies. Until their abnormal pregnancy methodology can be enhanced as described, the manuscript is best served by confining itself to observations across gestation in normal healthy pregnancies.

We thank the reviewer for this comment. As mentioned in response R3.1 we have now confined ourselves to PE patients (with or without FGR) to try to help to address these comments. We have more carefully defined the compromised pregnancies and have added further data where possible to support their inclusion (see response R3.1). Unfortunately, there was no funding in this project for maternal blood sampling and, whilst we accept that PlGF has been recommended to rule out pre-eclampsia in the UK by NICE, it is not routinely measured in the tertiary NHS hospital that these patients attended. This is a limitation of these results, which we have acknowledged and discussed on page 12. UtAD is also not routinely performed.

R3.5 The investigators identify typical intra-placental maternal blood flow patterns that are readily seen using high resolution gray-scale ultrasound - for example identification of the physiologic marginal sinus, though using color Doppler, flow within these sinuses is typically <10cm/sec. I agree that maternal blood may collect, and flow slowly, under regions of the chorionic plate, or flow as channels between groups of placentomes, but these are minor variations of normal. PlGF levels are normal in such circumstances (unpublished data).

We agree with the reviewer that the vasculature supporting the placenta can be studied on Doppler USS. However, we have not focused on the marginal sinus in this paper and in fact our ROIs do not specifically include it. In response to this comment we plan to consider the sinus more specifically in future experiments. Our primary focus here is on the placenta (as we have now stressed on page 3) and so our velocity sensitivity was set accordingly very low. Ultrasound can detect jets adjacent to the spiral arteries using low pulse repetition rates but only within ~1 cm of the endometrium where net flows are relatively fast, as highlighted in [5]. The coherent flow velocities measured by ultrasound in the large vessels are indeed much higher than we detect in the placenta where the inflow distributed over a much larger volume. In the wall, we can only measure net flows within the relatively large MRI voxels which will be less than local peaks within the vessels.

We believe that the IVIM measure is very similar to the signature detected by power Doppler ultrasound. A systematic comparison of the two techniques would probably be interesting.

3.6 I agree that the uterus is a fistula, however most blood flows from the uterine arteries to the ovarian veins, directly via the myometrium. The fraction of blood than enters the utero-placental arcuate arteries, and is directed to the spiral arterioles, is small, and is then rendered capable of entering the tier-villous space exploiting the Ventouri effect, to slow blood flow velocity and pressure, so that maternal perfusion does not compress the distal terminal villi that perfuse fetal bold at low pressure (see Burton G et al., Placenta 2009). So page 6 lines 1-2 findings may be explained by lack of normal spiral artery formation.

We completely agree with this analysis, and have tried to clarify the text where we make this point on page 4 and 11. We have also included these additional references in the description of the healthy placenta also on page 4.

3.7 Figure 3 cannot be interpreted as the type of specific placental disease is not presented. These diseases vary so much that it conveys no useful meaning to group these "compromised" cases together into one group. Yes some placental dysfunction can be characterized by "placental hyperinflation" (see Porat S et al., Ultrasound Obstet Gynecol 2013) or may be enlarged by blood [Breus' Mole; see Alanjari A et al] or by chorioangiosis of maternal or fetal anemia - all of which increase maximum placental thickness.

We hope we have addressed this comment by focusing on subjects with pre-eclampsia (see R3.1). We have also added a new discussion about placental thickness on page 9. Interestingly when split like this there is a difference in kurtosis depending on pre-eclampsia and birthweight, consistent with reduced villous branching in FGR but we have not added this to the paper as numbers are so small in this case:

3.8 I can see that the upper and centre R panels of Figure 3b show a pathologically thicker placenta (contrasts well with figures 3 L upper and middle of the normal imaging - what is the cause in these specific cases? That is why placental pathology is so important for interpretation. Please review Kingdom J et al., AJOG 2018 review article of imaging/pathology to then consider what type of placental disease you are imaging.

We have now included placental thickness to length ratio in supplementary materials (Table S2a and Figure S2) as we agree that this provides an additional method of characterizing compromised pregnancies. We have also added a comment on p9.

R3.9 This manuscript as presented does not discuss much of the pertinent literature summarized in this review.

Thank you for highlighting these interesting papers, which we have now added to the manuscript.

R3.10 2D ultrasound has thus far been seen to be capable of making a prenatal diagnosis of various placental diseases.

We absolutely agree that ultrasound is a very powerful clinical imaging technique in pregnancy. Here we are aiming at improved understanding of physiology rather than direct diagnosis. We have altered the last 2 paragraphs of the introduction to stress this on p3 and 4.

R3.11 Figure 4c panel 5 ; the arrows are not clear (as white), but the findings are novel as they show marginal blue signals (blood leaving via the marginal sinus) and capture the red blush of blood entering the inter-villous space via a presumed spiral artery. Therefore the entirety of the red blush represents a single placentome, since placentomes are perfused centrally.

Thank you for this important observation. We hope you do not mind but we have borrowed your compelling language in our description of the placental oxygenation data (page 8).

This comment also inspired us to look further at the area around the marginal sinus. The data suggests there may be a difference in the rate at which high flow is observed at the margins of the placenta in PE pregnancies. However, we need to do a more complete analysis, probably including a wider group of subjects to be sure and so we have not included this in the revised paper at this stage (see response to R3.5).

R3.12 Placental pump. In figure 5, the inferior myometrium is thicker on the R panel than on the L, which implies a Braxton-Hick's contraction. BH contractions can substantially distort the normal placenta. I have no problem with the BH causal interpretation, but I would not call it a placental pump.

We confused matters by describing this phenomenon as the ‘placental pump’. What we observe is a thickening and shortening of the uterine wall ONLY under the placenta (whilst the rest of the uterus actually expands) and a simultaneous reduction in the volume of the placenta. The data shows the wall contracting under the placenta but not across the whole uterus. In order to illustrate the phenomenon more clearly we have added videos showing the contraction (Figure S3a-b). We consider Braxton Hicks contractions to involve the whole uterus, and have identified a few such contractions in the dataset. An example is shown in figure S3c-d and this contraction has no significant effect on the placental volume. The text has been changed on page 9. We have now renamed the phenomenon the *uteroplacental pump* but we welcome any other suggestions that would improve clarity.

R3.13 Yes the proximal villous arterial trees (generations 1-3) have microscopic vertically-oriented contractile myofibroblasts, but the circumferential fibres dominate, and also the vast majority of these are not connected to the anchoring villi; rather, they are used to position the free-floating villi. Mechanically, they could not have the force needed to control the size of the IVS vs the myometrium. The anchoring villi are critical for preserving placental shape, as described in Porat S et al, 2013 (and illustrated in Kingdom J AJOG 2018). The villous abnormality of distal villous hypoplasia means there are much fewer anchoring villi, and in MVM pathology there can be accompanying decidual necrosis (laminar necrosis) ; so the scarce anchoring villi in this state rupture over time - and as such "placental hyper-inflation" evolves during the 2nd trimester, and leads to severe fetal growth restriction (and chronically-low PlGF - unpublished). Therefore the investigators are advised to remove the concept of a placental pump as the data are not presently convincing.

Once again we agree that the term ‘placental pump’ was confusing since it implied that we were suggesting that the observed compression of the placenta was driven primarily by contraction of the villi; from the videos (Fig. S3a-d) it is clear that it largely results from a local contraction of the myometrium of the placental bed (and relaxation or at least expansion elsewhere; added text page 11). We are aware of the contractile elements within the villi but we believe that these will simply prevent the IVS from increasing in thickness in response to contraction of wall. We note work in electrophysiology in rats has recently identified a ‘myometrial placental pacemaker zone’ and has noted that potentials related to contractions tend to initiate either at placental sites on the mesometrial border, or near the ovarian end [6]. In the light of the reviewers’ comments we have renamed this the uteroplacental pump. We hope that the included videos will demonstrate the phenomenon more clearly.

R3.14 Another cause of episodic placental hyperinflation is intermittent occlusion of utero-placental veins by placental villi, originally described by Kaufmann P. We have seen dramatic examples of episodic placental hyper-inflation, acting like a toilet cistern; as the IVS expands due to venous occlusion, finally the occluding villi are pulled out of the veins, and the whole IVS then empties again. I have seen this cycle occur over about 20 mins; it must be rare, I have seen this myself only 2-3 instances over many years, whereas BH contraction distortion of placental morphology is very common and is a confounder of the screening utility of placental morphology in clinical practice.

This is a very interesting observation. We have reviewed our data, and as far as we can tell we are not seeing any increases in volume prior to the contraction so we do not believe that this is the origin of the placental contractions that we are reporting but we will keep a look out for this phenomenon in future.

R3.15 In summary, I believe this is an important and novel manuscript, if it is confined to physiology and physiology variants. The team are strongly encouraged to strengthen their link between imaging and pathology, any including MFM and perinatal-placental pathology as described above, so as to build on the suggested edited manuscript, and subsequently generate clinical-MRI-pathology data that is much more compelling mechanistically.

Thank you for your encouraging comments. Your comments have greatly strengthened our interpretation of the data. We have narrowed our clinical group to only include PE using the histology available to confirm the definition of the group. A detailed comparison of histology and in vivo flow and oxygenation is our next goal but would require a very different study, focusing on detailed scanning in individuals close to planned delivery.

**References for this review**

1.	Jain V, Abdulmalik O, Propert KJ, Wehrli FW. Investigating the magnetic susceptibility properties of fresh human blood for noninvasive oxygen saturation quantification. Magn Reson Med [Internet]. 2012;68(3):863–7.

2.	Sun LQ, Macgowan CK, Portnoy S, Sled JG, Yoo SJ, Grosse-Wortmann L, et al. New advances in fetal cardiovascular magnetic resonance imaging for quantifying the distribution of blood flow and oxygen transport: Potential applications in fetal cardiovascular disease diagnosis and therapy. Echocardiogr J Cardiovasc Ultrasound Allied Tech. 2017;34(12):1799–803.

3.	Portnoy S, Milligan N, Seed M, Sled JG, Macgowan CK. Human umbilical cord blood relaxation times and susceptibility at 3 T. Magn Reson Med [Internet]. 2017;

4.	Sørensen A, Hutter J, Seed M, Grant PE, Gowland P. T2* weighted placental MRI: basic research tool or an emerging clinical test of placental dysfunction? Ultrasound Obstet Gynecol [Internet]. 2019;uog.20855.

5.	Collins SL, Stevenson GN, Noble JA, Impey L. Developmental changes in spiral artery blood flow in the human placenta observed with colour Doppler ultrasonography. Placenta. 2012;33(10):782–7.

6.	Lutton EJ, Lammers WJEP, James S, van den Berg HA, Blanks AM. Identification of uterine pacemaker regions at the myometrial-placental interface in the rat. J Physiol [Internet]. 2018;596(14):2841–52.
