# Supplementary material for: The haemodynamics of the human placenta in utero
Source: PLoS Biol. 2020 May 28;18(5):e3000676. doi: 10.1371/journal.pbio.3000676 (PMC7255609; doi:10.1371/journal.pbio.3000676)
Supplement: Attachment — Submitted filename: Response 20Mar20.docx [file pbio.3000676.s019.docx]

**Reviewer #1:**

*R1.1 Lack of histopathological correlations (except for four participants, listed in Table S2a).*

As we tried to explain previously, unfortunately the placentas were sent for histological examination in only four participants, so there is no opportunity to provide histology on the other subjects. Current practice is for histological examination to be undertaken in women with preterm pre-eclampsia, whose babies are admitted to the NNU although at times this is not prioritised over other clinical work. Term placentas are not routinely sent for histological examination, unless there is a major complication at delivery. Ideally we would have arranged to store all the placentas from all these pregnancies but that would have required us to have appointed a member of staff (probably clinically trained) to be present at the time of all deliveries, which was not possible within the funding available.

However the literature consistently shows differences in histology of these two groups of subjects, with pathophysiology (placental MVM) seen in the early onset placenta. The 4 samples analysed by the pathologists in this study did show these histopathological changes.

Reviewer 3 (R3.4) has recommended that such information is collected in future and we have adjusted our discussion in line with his comments to ensure future readers realise the importance of such data.

*R1.2 The authors did not really address my comment regarding Fig. 2C, and in the revised version (page 4) they have removed any text reference to Fig. 2C.*

Unfortunately, this has gotten a little confusing as Figure 2c in the original submission is now Figure 2b. However, the reviewer is right that we had failed to discuss current Figure 2c in the text. We apologise for this oversight which was caused by Figure 2c being simply an illustration of a method, but we have now referred to it properly in the text. We have also further reviewed and revised the discussion of Figure 2b in case the reviewer was also referring to that figure.

*R1.3 While the paper is indeed important, greater attention to readability and flow can improve the revised text.*

We have taken time to edit the paper to try to improve readability.

**Reviewer #3**

*R3.1 I remain skeptical about your proposed concept of a utero-placental pump, but I'm open-minded enough to allow the reader to decide. I spend a lot of time scanning pregnancies with complex placental problems, and focal (non-concentric) uterine contractions are common; they can very easily lead to mis-interpretation via placental distortion, so its important to re-assess the placenta when the uterus is soft and the myometrium is uniform.*

We are grateful that the reviewer is open minded about this. We would highlight that we do observe the placenta relax after a contraction. We have added this to the manuscript on p10.

*R3.2 Some investigators have previously reported that placentas of near-term pregnancies with preeclampsia are heavier than average, and increased central placental thickness has been reported in the 1st trimester (NT scan) examination, in women who subsequently develop preeclampsia, and this phenomenon persists in the 2nd trimester.*

This agrees with our data in Figure S2.

*R3.3 Most women who develop preeclampsia at term (37+ weeks) have no demonstrable placental disease ; rather, they have "maternal" disease, as opposed to "placental MVM" disease (eg see prospective cohort study, Wright E, & Kingdom J, Obstet Gynecol, Nov 2017). They have well-perfused placentas with normal uterine artery Doppler, and so do not have hypopxia-reoxygenation injury (which represses PlGF production) as is found in the preterm preeclamptic placenta, with histologic features of MVM including deciudual vasculopathy. That is why maternal PlGF testing is only valid up until 36 weeks' gestation.*

We agree. However all our women with PE had onset before 36 weeks.

*R3.4 I hope you include PlGF testing and placental pathology costs in your future grant applications, as they are cost-effective and important phenotypic tests.*

We agree these important measures should be included in future studies. We have further stressed this in the discussion.
